# Dysfunctional Activation and Brain Network Profiles in Youth with Obsessive-Compulsive Disorder: A Focus on the Dorsal Anterior Cingulate during Working Memory

**DOI:** 10.3389/fnhum.2015.00149

**Published:** 2015-03-17

**Authors:** Vaibhav A. Diwadkar, Ashley Burgess, Ella Hong, Carrie Rix, Paul D. Arnold, Gregory L. Hanna, David R. Rosenberg

**Affiliations:** ^1^Department of Psychiatry and Behavioral Neurosciences, Brain Imaging Research Division, Wayne State University School of Medicine, Detroit, MI, USA; ^2^Department of Psychiatry, Hospital for Sick Children, University of Toronto, Toronto, ON, Canada; ^3^Department of Psychiatry, University of Michigan, Ann Arbor, MI, USA

**Keywords:** dorsal anterior cingulate cortex, obsessive-compulsive disorder, network analysis, working memory, fMRI

## Abstract

Brain network dysfunction is emerging as a central biomarker of interest in psychiatry, in large part, because psychiatric conditions are increasingly seen as disconnection syndromes. Understanding dysfunctional brain network profiles in task-active states provides important information on network engagement in an experimental context. This in turn may be predictive of many of the cognitive and behavioral deficits associated with complex behavioral phenotypes. Here we investigated brain network profiles in youth with obsessive-compulsive disorder (OCD), contrasting them with a group of age-comparable controls. Network interactions were assessed during simple working memory: in particular, we focused on the modulation by the dorsal anterior cingulate cortex (dACC) of cortical, striatal, and thalamic regions. The focus on the dACC was motivated by its hypothesized role in the pathophysiology of OCD. However, its task-active network signatures have not been investigated before. Network interactions were modeled using psychophysiological interaction, a simple directional model of seed to target brain interactions. Our results indicate that OCD is characterized by significantly increased dACC modulation of cortical, striatal, and thalamic targets during working memory, and that this aberrant increase in OCD patients is maintained regardless of working memory demand. The results constitute compelling evidence of dysfunctional brain network interactions in OCD and suggest that these interactions may be related to a combination of network inefficiencies and dACC hyper-activity that has been associated with the phenotype.

## Introduction

Obsessive-compulsive disorder (OCD) is a commonly occurring childhood and adolescent-onset neuropsychiatric disorder. It is characterized by obsessions (recurrent and persistent thoughts that typically induce marked distress) and compulsions (repetitive behaviors aimed at alleviating distress). OCD represents the upper extreme of an underlying continuous trait distribution encompassing obsessive-compulsive behaviors common in the general population that are heritable and cross traditional diagnostic boundaries. Thus, OCD represents a clinical “end-point” for a commonly observed trait (~45% of adolescents report OCD symptoms) (Berg et al., 1988; Apter et al., 1996). The 1-year incidence of OCD and sub-clinical OCD in adolescents is ~0.7 and 8.4%, respectively (Valleni-Basile et al., 1996). These relatively high rates of incidence and the association with a trait evident in the general population emphasize the importance of characterizing biological mechanisms underlying OCD. In this report, we aim to characterize these biological mechanisms by investigating brain network interactions in OCD and their differences from typical healthy controls.

Understanding brain network profiles and brain network dysfunction is a central theme of interest in clinical neuroscience. As suggested by the National Institute of Mental Health (Insel et al., 2010), such a focus may lead to an enhanced understanding of specific bio-behavioral impairments that underpin the emergence of complex behavioral phenotypes which are classified as psychiatric disorders. Indeed, understanding network dysfunction, in particular, is emerging as a leading framework for characterizing the neural substrates of multiple psychiatric conditions (Friston, 1998; Stephan et al., 2006; Almeida et al., 2009; Shaw et al., 2009; Diwadkar, 2012; Schmidt et al., 2013).

Obsessive-compulsive disorder, like most neuropsychiatric conditions, often has its origins in childhood and adolescence when brain network function is still maturing (Paus et al., 2008). Ensuing disordered neurodevelopmental trajectories (in the absence of adaptive responses) may in turn mediate the continued expression of symptoms through adolescence and into early adulthood (Tottenham and Sheridan, 2009). Furthermore, the complex patterns of OCD symptoms are linked to the inability to disengage behaviors from intrusive thoughts, implying aberrantly increased inhibitory control (Bari and Robbins, 2013). These patterns are highly suggestive of dysfunctions in control mechanisms within relevant brain networks (Piras et al., 2013). In this context, the role of the dorsal anterior cingulate cortex (dACC) assumes significance.

The dACC is positioned as a principal control region in the brain (Paus, 2001) that by itself, or through its mediation of cortical-striatal networks, exercises aspects of cognitive and motor control (Bakshi et al., 2011). The region has been of particular interest in OCD: glutamate dysregulation in the anterior cingulate and striatum has been implicated in pediatric OCD patients (Rosenberg et al., 2000, 2004). Altered glutamate concentrations may be linked to dysfunctional fMRI responses during tasks of behavioral engagement and disengagement. For instance, during conflict processing and action monitoring, OCD subjects evince higher activation in regions including the anterior cingulate cortex and the striatum (Fitzgerald et al., 2005; Maltby et al., 2005; Marsh et al., 2014) that may provide functional expressions of dACC dysfunction in the illness. A question of interest is whether these hyper-activations in the dACC are associated with dysfunctional network profiles.

Network models of fMRI have been applied in OCD. However, a principle focus of network-analyses of *in vivo* imaging data has been on the classification of resting state *functional connectivity* within (and across) cortical, limbic, striatal, and cerebellar networks (Harrison et al., 2009; Peng et al., 2014). These analyses have been notable as they have revealed categorical and developmental distinctions in resting state functional connectivity (rsFC) between OCD and typical controls in frontal, striatal and thalamic (FSTC) circuits (Fitzgerald et al., 2011). rsFC results are not directly informative about dysfunctional dACC-related profiles in a task-active state. For instance, the relationship between resting state functional connectivity (rsFC) and *task-dependent* functional interactions between regions remains uncertain (Stephan, 2004) and experimental analyses of within subject data have been equivocal (Rehme et al., 2013). Thus rsFC and the low-frequency bold signals it correlates between provide a complimentary snapshot of pathology; task-active analyses of functional network interactions are important for assessing a measure of network dynamics. Moreover, a separate question of interest is whether dysfunctional activation and brain network profiles in OCD are observed in tasks *not involving conflict monitoring*. Such evidence will provide strong support for general network based dysfunction in the disorder extending beyond highly circumscribed behavioral domains.

**Figure 1:**
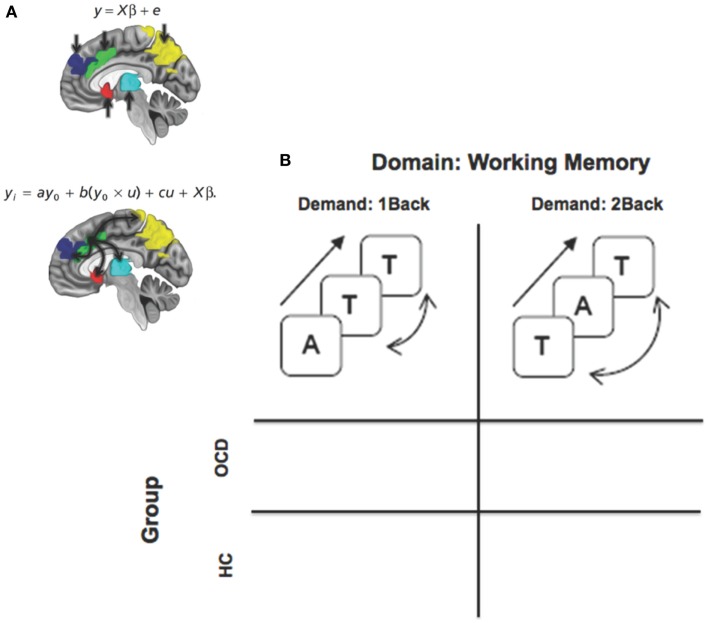
**A framework for assessing dysfunctional activation and dACC-related network profiles of cortical, striatal, and thalamic networks in OCD**. **(A)** The two panels depictive activation-based and seed-based approaches to identifying function and dysfunction. The equations represent basic linear model formalisms for each class of models. Note the convolution term (*y*_0_ × *u*) in the PPI based model that accounts for seed (*y*_0_ = dACC) modulation of targets in the task-oriented (*u* = working memory > rest) context. The regions of interest are schematically depicted on the mid-sagittal surface. The second figure schematically depicts the modulatory effects of the dACC assessed using psychophysiological interaction. **(B)** The factorial design space used for the study that assessed the effects of task-demand (1Back vs. 2Back) crossed with group.

We had two principal aims in this study (summarized in Figure 1): (a) to investigate network profiles originating in the dACC in the task-active state using psychophysiological interaction (PPI) (Friston et al., 1997; O’Reilly et al., 2012), PPI is a simple framework within the general linear model for investigating contextual modulation of targets (e.g., regions within FSTC) by a seed (e.g., dACC) in a task-active context; (b) to investigate these profiles during parametrically manipulated verbal working memory, (Casey et al., 1995; Diwadkar et al., 2011, 2013), a domain that provides a rich window for investigating normal and dysfunctional activation and network profiles in the FSTC.

## Materials and Methods

### Participants

Eighteen participants with a diagnosis of OCD and 27 controls participated in the fMRI studies (see Table 1). All participants and their parents were interviewed with the Schedule for Schizophrenia and Affective Disorders for School-Aged Children-Present and Lifetime Version and Schedule for Obsessive-Compulsive and Other Behavioral Syndromes (Wolff and Wolff, 1991; Kaufman et al., 1997). The lifetime (maximum) and current severity of OCD were assessed in the patients with a modified version of the Children’s Yale-Brown Obsessive Compulsive Disorder Scale (Goodman et al., 1989; Scahill et al., 1997). Lifetime and current axis I diagnoses were made independently by two clinicians (David R. Rosenberg, Gregory L. Hanna) using all sources of information according to DSM-IV criteria. All patients with OCD had a total lifetime CY-BOCS score of at least 20. Exclusion criteria for patients and controls included lifetime history of psychosis, bipolar disorder, substance abuse or dependence, anorexia or bulimia nervosa, epilepsy, head injury with sustained loss of consciousness, Huntington’s disease, dyskinesia, chronically disabling medical illness, autism, mental retardation, or a score >15 on the lifetime version of the Social Communication Questionnaire. Controls were free of all psychiatric illness. Legal guardians provided written informed consent prior and children gave written assent prior to participating in the study. The Human Subjects Investigative committee at Wayne State University and the University of Michigan approved the protocol and all methods therein.

**Table 1:** **The table depicts the demographics for healthy control (HC) and OCD participants**.

	M/F	Mean age	Range	Height (inches)	Weight (lbs)	Handedness (R/L/M)	CY-BOCS (*T*)	CY-BOCS (*O*)	CY-BOCS (*C*)
Typical controls (*n* = 27)	18/9	17.4 (3.14)	12–21	67.3 (4.8)	147.8 (52.9)	24/2/1			
OCD (*n* = 18)	11/7	17.2 (3.33)	11–21	65.6 (4.3)	146.3 (60)	17/1/0	31/16 (4.5/9.4)	15/8 (2.7/4.8)	16/8 (2.7/4.9)

*Groups did not differ in terms of age (*t* = 0.32, *p* = 0.75), height (*t* = 1.22, *p* = 0.23), or weight (*t* = 0.08, *p* = 0.94). Also comparable were gender (*χ*^2^ = 0.15, *p* = 0.70) and handedness frequencies (*χ*^2^ = 0.76, *p* = 0.68). Values in parenthesis represent SD. For lifetime CY-BOCS (lifetime/current): *T* = Total symptoms, *O* = Obsessive Symptoms, *C* = Compulsive symptoms*.

### fMRI

Gradient echo EPI fMRI data acquisition was conducted at Vaitkevicius Magnetic Resonance Centre on a 3T Siemens Verio system using a 12-channel volume head coil (TR: 2.6 s, TE: 29 ms, FOV: 256 mm × 256 mm, acquisition matrix: 128 × 128, 36 axial slices, voxel dimensions: 2 mm × 2 mm × 3 mm). In addition, a 3D T1-weighted anatomical MRI image was acquired (TR: 2200 ms, TI: 778 ms, TE: 3 ms, flip-angle = 13°, FOV: 256 mm × 256 mm, 256 axial slices of thickness = 1.0 mm, matrix = 256 × 256). A neuroradiologist reviewed all scans to rule out clinically significant abnormalities.

During fMRI, subjects were positioned with adjustable padded restraints employed for head stabilization. Stimuli were rear-projected using an IFIS-SA presentation system (MRI Devices), and subjects responded with a button box unit. During fMRI, subjects participated in an established verbal *n*-back paradigm (Casey et al., 1995). Parametric working memory load was varied between maintaining 0, 1, or 2 items in memory (0-, 1-, or 2-Back; see Figure 1 insets). Runs were blocked by condition. During each block (30 s), letters were projected in sequence (presentation time: 500 ms; ISI: 2500 ms; 10 letters per block) on a screen; subjects signaled with a two-choice optical response box if the presented letter was a target or not. The paradigm cycled between rest (20 s), 0-, 1-, and 2-Back epochs (three blocks each). The experiment was controlled using presentation (Neurobehavioral Systems Inc.).

### fMRI Processing

fMRI data were processed in SPM8 using typical methods. All images were manually oriented to the AC-PC line, realigned to correct for head movement, spatially normalized to the MNI (Montreal Neurological Institute) template brain and resliced (2 mm × 2 mm × 2 mm). Low frequency components were removed using a low-pass filter (128 s) and images were spatially smoothed using a Gaussian filter (8 mm full-width half maximum; FWHM). An autoregressive AR(1) model was used to account for serial correlation, and regressors modeled as a 30 s boxcar vectors (for each of the task-related conditions) were convolved with a canonical hemodynamic reference waveform.

Subjects’ head motion was within accepted limits (<4 mm). Furthermore, in all first level models, the effects of motion were modeled by including the six motion parameters as covariates of no interest. First-level contrasts (1Back > 0Back; 2Back > 0Back) were used to assess the effects of memory load on activation.

PPI (implemented in SPM8) was employed to model dACC modulation of FSTC targets during working memory (Friston et al., 1997; Honey et al., 2005). For *each subject*, time series from the effects of interest contrast (*p* < 0.05) were extracted from the dACC peak (including Brodmann areas 32 and the supra-genual aspects of BA24) (Palomero-Gallagher et al., 2008). The extracted time series (the wave form of which provides an estimate of the continuous physiological response of the dACC) was subsequently convolved with the two contrasts of interest reflecting effects of differential memorial load, specifically, 1-Back > 0-Back (low load) and 2-Back > 0-Back (high load). The resultant interaction term was positively weighted to assess the facilitating influence of the dACC on FSTC targets (with the slope of the effect parametrically encoded in the convolution term and reflecting the degree of modulation).

For all activation or network analyses, first level maps (activation or PPI) from each subject were submitted to a second-level random effects analyses of variance with group modeled as an independent factor and memory load as non-independent factor. The factorial design permitted assessment of intra-group load-related effects, as well as between-group differences at varying levels of memory load.

All second level analyses were spatially thresholded in the FSTC regions of interest using deterministic anatomical masks defined in stereotactic space (Maldjian et al., 2003). These maps constitute anatomical representations in stereotactic space representing each of the regions of interest and are widely employed to spatially localize activations in neuroimaging research. Images were corrected using cluster level correction (cluster extent thresholds, *p*_c_ < 0.05) derived from 10^4^ Monte Carlo simulations from voxels across the individual regions of interest (Ward, 2000). Individual voxel peaks in significant clusters are reported in terms of Montreal Neurological Institute coordinates.

## Results

Results are organized to sequentially present evidence of (1) dysfunctional activation profiles and (2) dysfunctional brain network profiles in OCD and HC:

(1a) We first show load-related effects on *activation profiles* within both HC and OCD. These results provide evidence of *within-group* effects of parametric increases in working memory load on FSTC.
(1b) Next we present *between-group* results showing aberrantly increased *activation profiles* in FSTC in OCD patients compared to HC at both levels of memory load. These results demonstrate that OCD participants more extensively activate FSTC than HC at both levels of memory load.
(2) We show between group results assessing dysfunctional brain network profiles in OCD compared to HC. These results indicate aberrantly increased modulation of FSTC by the dACC in OCD, especially at the lower level of memory load.

### Load-related effects on activation profiles

**Figure 2:**
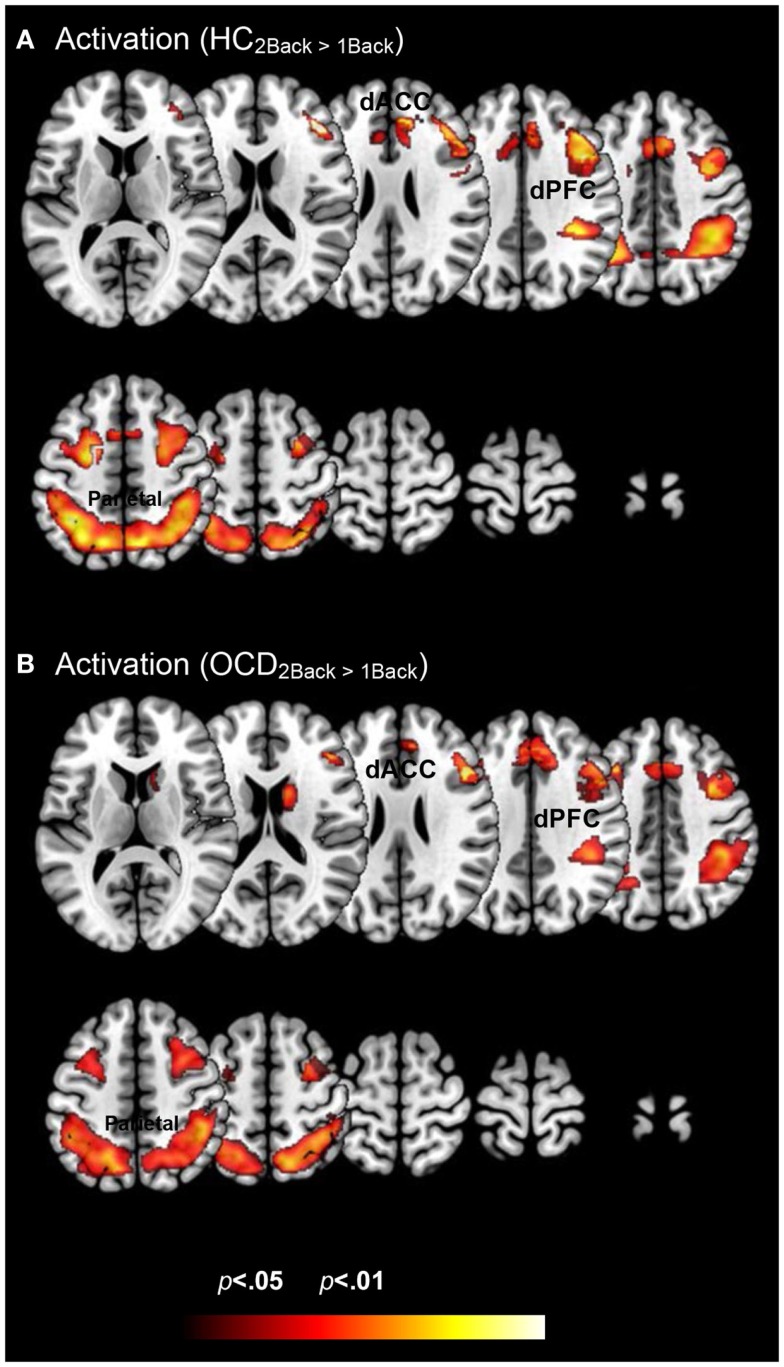
**Within group changes in activation profiles as a function of load are depicted on identical ascending mosaics of axial views**. The significant clusters (*p* < 0.05, cluster level) show significant increases in activation with increases in working memory related load. As seen, these increases are evident within both **(A)** healthy control and **(B)** OCD groups. These activation profiles establish within group effects of memory load across previously implicated load sensitive working memory related regions. These include dorsolateral prefrontal cortex (dPFC), the dorsal anterior cingulate (dACC), and the parietal cortex.

Figure 2 depicts clusters (*p*_c_ < 0.05) in FSTC showing increased within-group activation in response to increases in memory load (cluster relevant information in Table 2). In both groups, increased memory load results in increased recruitment of frontal and parietal regions, and the dACC. These results are unsurprising for the HC group. They are highly consistent with previous assessments of activation profiles in this circuit in HC (Braver et al., 1997; Cohen et al., 1997; Diwadkar et al., 2000), showing increased recruitment in brain circuits committed to implementing working memory related functions. The results in OCD are notable as they demonstrate that the memory effect exerts within-group effects consistent with HC. This is important evidence that FSTC in OCD is sensitive to load-related variations in working memory and that the overall implementation of the task generates load-related effects on activation profiles. Notable is an absence of load-related activation effects in the striatum or the thalamus, regions not typically implicated in core memory-related processing. The basal ganglia contribute to cortical-striatal processing loops that sub-serve complex processing, by supplementing prefrontal function (Hazy et al., 2006; Calzavara et al., 2007; Voytek and Knight, 2010). The thalamus forms cortical-thalamic processing units that integrate information from cortical and striatal loops to modulate complex behavior, but has generally not been sensitive to load-related variations in working memory (Haber and Calzavara, 2009).

**Table 2:** **The table provides information on clusters of significance and peaks within where each of the groups showed increased *activation* to variations in memory load (Figure 2)**.

Region	Brodmann area	MNI coordinates (*x*, *y*, *z*)			*Z* score	Cluster extent	*p* (peak)
**Table 2: activation**
HC_2Back > 1Back_
Parietal lobe	40	−36	−51	49	5.23	734	0.000
Mid frontal gyrus	8	27	15	45	1.93	135	0.027
Dorsal prefrontal cortex	46	−42	20	27	4.45	74	0.000
Basal ganglia	−	28	18	6	3.37	32	0.000
dACC	24	−18	−1	51	4.42	177	0.000
OCD_2Back > 1Back_
Parietal lobe	40	46	−40	51	4.08	739	0.000
Mid frontal gyrus	6	32	8	52	3.17	175	0.001
Dorsal prefrontal cortex	9	−39	12	39	3.08	103	0.001
Basal ganglia	−	14	6	19	3.37	70	0.000
dACC	32	8	20	46	3.19	189	0.001

### Between-group results showing aberrantly increased activation profiles in FSTC in OCD patients

**Figure 3:**
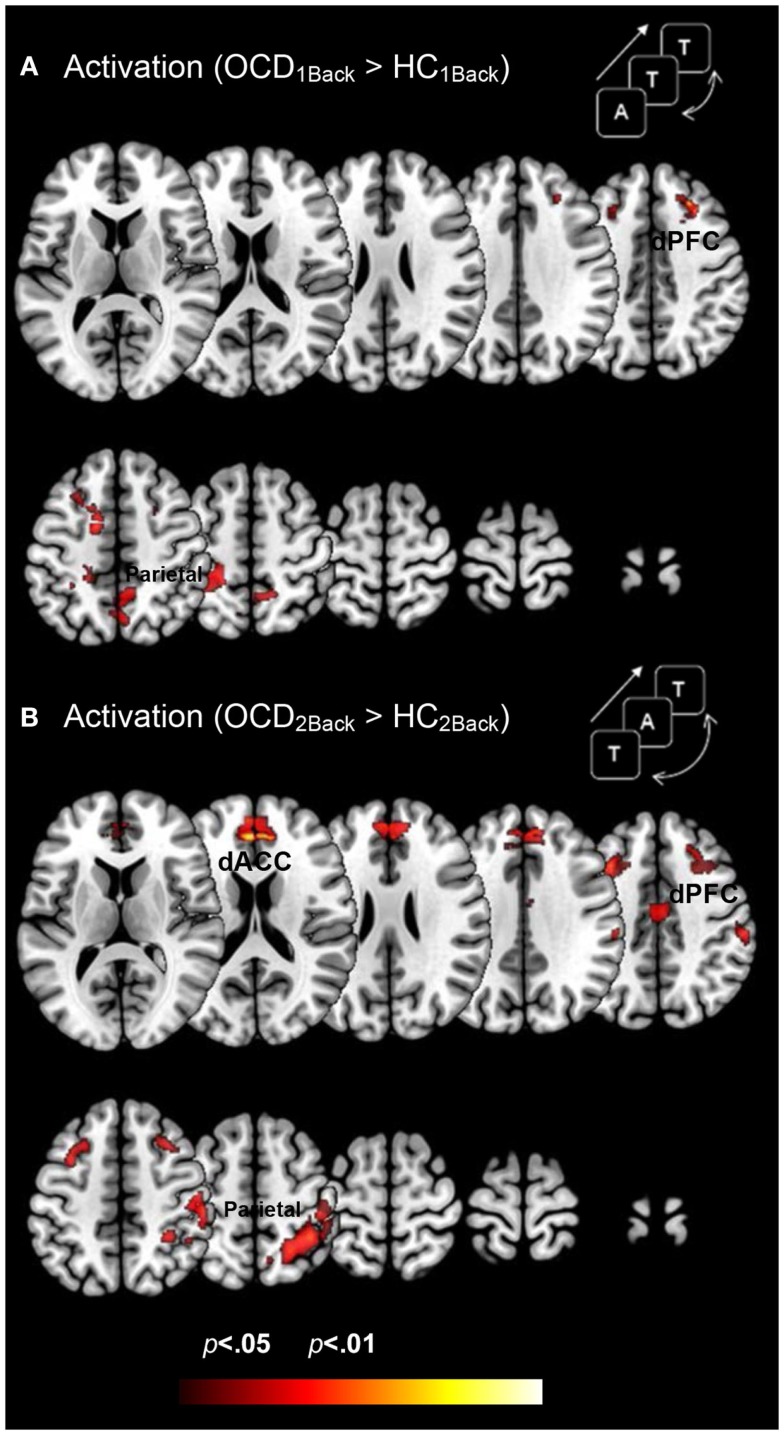
**Dysfunctional activation profiles in OCD (relative to controls) are depicted for both (A) the 1Back level of memory and (B) the 2Back level of memory load**. Increased activation in OCD (*p* < 0.05, cluster level) is depicted on identical ascending mosaics of axial views. These activation profiles indicate increased activation in dorsolateral prefrontal cortex (dPFC), the dorsal anterior cingulate (dACC), and the parietal cortex in OCD. Notably the degree of dysfunctional activation in OCD scales as a function of memory load. We speculate that the parametric demands as expressed in dysfunctional activation profiles load disproportionately in OCD participants. As will be seen, brain network profiles in OCD do not strictly follow activation patterns, evidence that signatures of network interactions may complement psychopathology revealed in activation models.

Figure 3 depicts clusters (*p*_c_ < 0.05) in FSTC showing increased activation in OCD (relative to HC) at each level of memory load (cluster relevant information in Table 3). Several effects are evident. Dysfunctional activation profiles are observed in the frontal and parietal cortices and in the dACC at both levels of load. Absent is evidence of dysfunctional activation profiles in the striatum or the thalamus. Moreover, dysfunction in activation profiles scales as a function of memory load: Increased memory demand leads to increased activation in cortical regions. These analyses are consistent with previous studies in FSTC in OCD participants in other behavioral domains such as conflict monitoring that are closely associated with behavioral phenotypes in the illness (Huyser et al., 2011). As one of our study aims was to assess whether hyper-activation in FSTC constitutes a domain-general property of brain regions in OCD, these analyses extend the findings beyond the domain of conflict processing and suggest that multiple tasks engaging FSTC are sensitive for detecting activation-related dysfunction.

**Table 3:** **The table provides information regarding clusters of significant and significant peaks showing *dysfunctional activation profiles* in OCD (compared to HC) at each level of memory load (Figure 3)**.

Region	Brodmann area	MNI coordinates (*x*, *y*, *z*)			*Z* score	Cluster extent	*p* (peak)
**Table 3: activation**
OCD_1Back_ > HC_1Back_
Parietal lobe	5	−18	−40	61	3.05	385	0.001
Mid frontal gyrus	8	27	27	42	2.45	83	0.007
Dorsal prefrontal cortex	9	24	38	36	2.8	58	0.003
dACC	24	−15	−1	49	2.87	117	0.002
OCD_2Back_ > HC_2Back_
Parietal lobe	3	50	−22	56	3.18	392	0.001
Mid frontal gyrus	6	38	21	45	3.03	124	0.001
Dorsal prefrontal cortex	9	9	47	33	3.07	97	0.001
dACC	32	−3	42	18	2.78	197	0.003

### Between group results assessing dysfunctional brain network profiles in OCD

**Figure 4:**
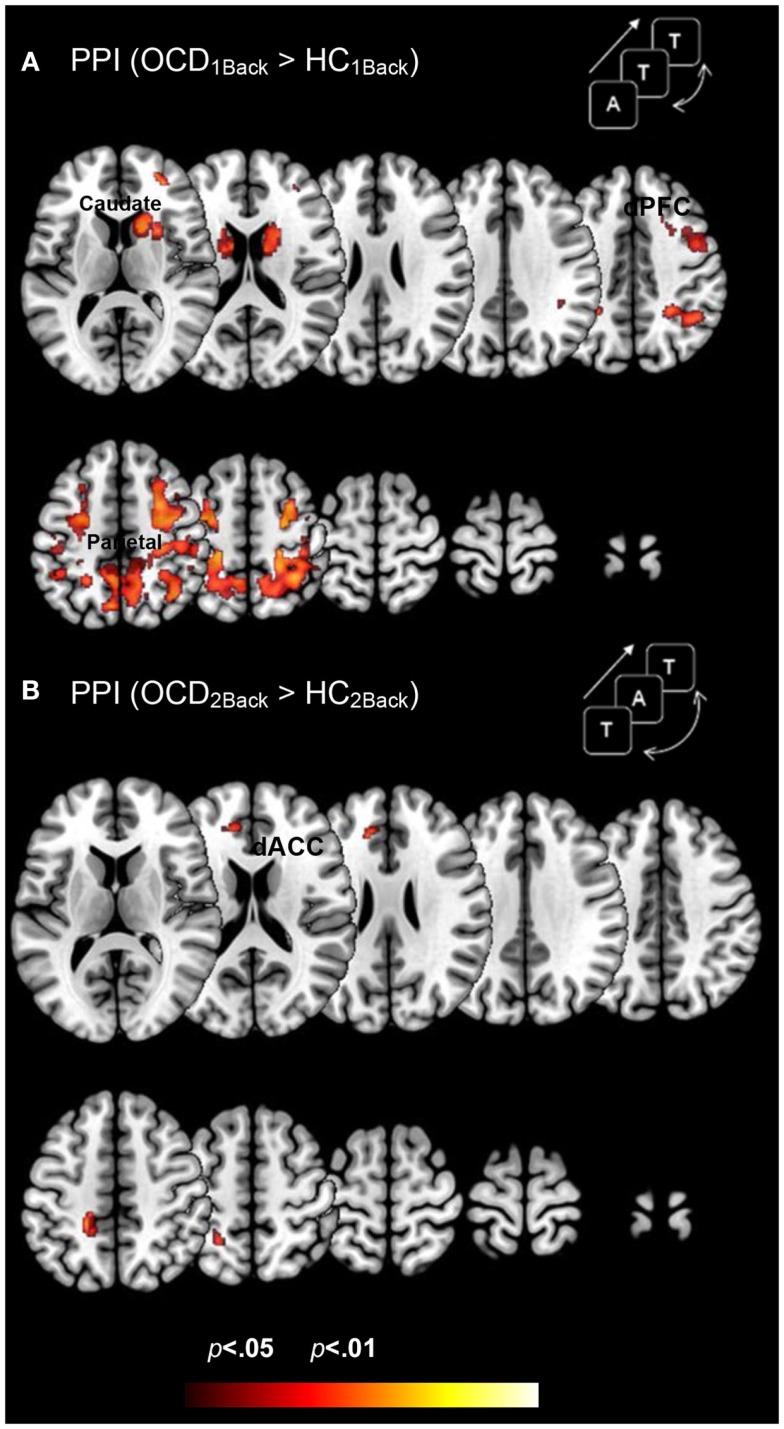
**Dysfunctional brain network profiles in OCD (relative to controls) are depicted for both (A) the 1Back level of memory and (B) the 2Back level of memory load**. The clusters depict significantly increased dACC-modulation of cortical and striatal targets in OCD compared to typical controls (*p* < 0.05, cluster level) depicted on identical ascending mosaics of axial views. These brain network profiles complement dysfunctional activation profiles (Figure 3). Note the implication of the caudate, not implicated in dysfunctional activation. The increased modulation by the dACC may reflect increased control-related inputs demanded in OCD to sub-serve network function associated with this fundamental domain. The lack of a parametric effect may reflect the fact that dACC related network engagement is already aberrantly increased at the 1Back level. Indeed, OCD participants did not show an increase in dACC modulation going from the 1Back to the 2Back level of demand (whereas HC participants did).

Figure 4 depicts clusters (*p*_c_ < 0.05) in FSTC showing *increased modulation by the dACC in OCD* (relative to HC) at each level of memory load (cluster relevant information in Table 4). We highlight several notable effects. First, dysfunctional network profiles in OCD form a pattern that is distinct and complimentary to that observed in activation profiles. OCD is characterized by increased dACC related modulation at the 1Back level of load but not the 2Back level, suggesting that the degree of dACC modulation (and the mechanisms that can be inferred from it) do not scale with load. We speculate (see Discussion) that this effect may be related to aberrantly increased dACC modulation at the 1Back level itself. The hyper-modulation may reflect inefficiencies in control-related network function or hyper-activity of the dACC, or both. Second, dysfunctional modulation of the striatum is evident, with significantly increased dACC modulation of the caudate and putamen observed at the 1Back level (Figure 4A). This effect also constitutes a complementary pattern of dysfunction from activation in OCD where profiles in the striatum appeared normal (Figure 3).

**Table 4:** **The table provides information regarding clusters of significant and significant peaks showing *dysfunctional network profiles* in OCD (compared to HC) at each level of memory load (Figure 4)**.

Region	Brodmann area	MNI coordinates (*x*, *y*, *z*)			*Z* score	Cluster extent	*p* (peak]
**Table 4: PPI**
OCD_1Back_ > HC_1Back_
Parietal lobe	7	20	−57	60	3.36	529	0.000
Mid frontal gyrus	6	−32	−6	54	3.13	144	0.001
Basal ganglia	–	20	9	15	3	61	0.001
OCD_2Back_ > HC_2Back_
Parietal lobe	7	−18	−36	48	2.72	223	0.003
Dorsal prefrontal cortex	9	−12	36	22	2.76	89	0.003

## Discussion

We conducted a simple investigation of brain activation and network profiles in a group of OCD youth and age-comparable controls. Participants were assessed with fMRI using a simple working memory paradigm with variable demands (Figure 1). Three principle results are highlighted across both classes of analyses: *Activation Profiles*: (1a) Activation profiles were highly sensitive to increases in memory load within each group (Figure 2). (1b) Youth with OCD were characterized by aberrantly increased recruitment of frontal and parietal regions (but not striatal or thalamic regions) during both levels of working memory. The degree of hyper-activation scaled as a function of working memory demand (Figure 3). *Network Profiles*: (2) Compared to HC, youth with OCD were characterized by increased dACC modulation of frontal, parietal, and striatal regions, particular at lower levels of working memory load (Figure 4).

Taken together, these results establish that OCD is characterized by dysfunction in core FSTC regions, detectable using both activation- and network-based analyses of fMRI signals. We suggest that the network-based analyses are notable for being the first to demonstrate dysfunctional network signatures of the dACC, a region closely associated with OCD related pathophysiology. Moreover, these profiles observed using a basic working memory paradigm, suggest that FSTC deficits are a basic pathophysiologic mechanism underlying OCD, are detectable with a multiplicity of tasks, and affect frontal, striatal, and thalamic circuits. In the remainder of the paper, we discuss the putative mechanisms that may underpin these observations and the implications for OCD related pathology and function.

### Cingulate, frontal, striatal, and thalamic regions: A critical circuit sub-serving complex function

The regions targeted in this investigation collectively form core sub-circuits that implement function in a multiplicity of higher-order domains including working memory (Owen et al., 2005; Diwadkar et al., 2011), sustained attention (Fan et al., 2005; Langner and Eickhoff, 2013; Diwadkar et al., 2014), and cognitive control (Carter et al., 1999; Anderson et al., 2008). These functional sub-circuits are also underpinned by dense patterns of anatomical connectivity. The dorsal-prefrontal cortex and the basal ganglia share topographically mapped monosynaptic connections (Calzavara et al., 2007) that may explain co-activation patterns frequently observed in fMRI studies. Descending connections from cortical regions including the prefrontal cortex and sensory, motor, and frontal regions synapse on multiple thalamic nuclei including the ventral and posteromedial complexes (Ray and Price, 1993; Klein et al., 2010; Li et al., 2013) leading to the notion of “cortical-thalamic processing units” (Briggs and Usrey, 2008). The dACC is uniquely positioned from an anatomical standpoint, with connections to frontal and motor regions, to play a mediating influence in control related mechanisms (Paus, 2001). Each of these regions appears to be relatively specialized for highly sophisticated functions.

The dorsal-prefrontal cortex sub-serves working memory in multiple ways. Phasic activity in prefrontal neurons is strongly correlated with the temporary maintenance of memoranda in working memory (Vijayraghavan et al., 2007), suggestive of a direct link between neuronal responses and overt behavior. Moreover, the prefrontal cortex sub-serves goal-directed behavior in multiple domains (including working memory) through direct “command” signals to multiple cortical and sub-cortical targets (Crowe et al., 2013; Funahashi and Andreau, 2013). The anatomical positions of the basal ganglia allow the structure to receive inputs from multiple uni- and heteromodal regions (Ragsdale and Graybiel, 1990). Thus the structure serves as a critical node in multiple network pathways, playing executive and supporting roles in several behavioral domains. Along with the prefrontal cortex, the basal ganglia appear to exert attention-related modulation of working memory related function (Herrero et al., 2002; McNab and Klingberg, 2008). The thalamus is considered a principle gateway to the cortex (McAlonan et al., 2008), engaged in filtering of massive sensory inputs, particularly in the visual domain, and sending extensive outputs to cortical and sub-cortical regions (Haber and Calzavara, 2009). The structure also plays essential computational roles by integrating network activity essential for modulating behaviors. Many of the psychological domains that are underpinned by regional function are implicated in OCD. Thus pediatric OCD patients in particular show deficits in sustained attention (Lucke et al., 2014), executive function and working memory (Melloni et al., 2012), and cognitive control and metacognition (Koch and Exner, 2015). It is therefore not surprising that frontal, striatal, and thalamic circuits have been identified as central to potential interventions in OCD (Burguiere et al., 2015).

### Hyper-*activation* in OCD during working memory: Possible mechanisms and relationship with other disorders

Though working memory deficits are generally seen as secondary to the core pathology of OCD (Harkin and Kessler, 2011), our activation results provide good convergence with recent reports. Memory load-related hyper-activation in frontal-parietal regions has been proposed as an intermediate phenotype for OCD, where the hyper-activation has been labeled as compensatory (Nakao et al., 2009; Koch et al., 2012; de Vries et al., 2013). Effects on dACC activation have, however, been equivocal; previous studies have shown a reverse effect of complexity on dACC activation in OCD, with disengagement of the structure following load related effects. Nevertheless, our results provide good conceptual overlap with studies in pathology that have linked hyper-activation under conditions of task compliance with regional efficiency. This concept of inefficiency finds pronounced expression in the schizophrenia spectrum, where disease-related effects (that have been associated with dopamine dysfunction) are presumed to affect the “duty cycle” of task-relevant brain regions including the prefrontal cortex and the striatum (Callicott et al., 2003; Manoach, 2003; Jansma et al., 2004; Meisenzahl et al., 2007; Diwadkar et al., 2012). These inefficiencies might imply that neuronal pools (that form one electrophysiological origin of the fMRI signal) (Logothetis and Wandell, 2004) engage in excess excitatory firing responses when demand is exerted on FSTC. Moreover, inefficiencies provide a window into the “scalability” of brain regions in response to demand. In other words, the functioning limits of FSTC in OCD may be compromised such that excessive cognitive demand may stretch FSTC ability to sub-serve function. In this view, FSTC hyper-activation during working memory far, from being a peripheral correlate of OCD, is a central mechanism underlying the illness, and a primary intermediate phenotype as previously proposed (de Vries et al., 2013).

A parallel explanation for hyper-activity is that it reflects glutamate-related dysfunction that affects how relevant regions are recruited for a task (Wu et al., 2012, 2013; Stewart et al., 2013; Pauls et al., 2014). As a principle excitatory neurotransmitter, glutamate exerts substantial effects on brain function, particularly in the excitatory model. Glutamate dysfunction in OCD can alter the neurochemical-electrophysiological relationship that sub-serves BOLD-based activation. A more complete assessment of the Glutamate-fMRI relationship will require assessment of both classes of signals acquired within subjects. This is an ongoing endeavor in our studies that involves multi-modal acquisition of fMRI and MRS data within subjects.

### Hyper-modulation of FSTC by the dACC: Novel evidence of dysfunctional network profiles

Relatively few studies have assessed connectivity in the task-active state in OCD. Psychophysiological interactions provide a straightforward model of directional effects of seed regions on their targets in a task-related context, providing a window into network interactions. This window is considered intermediate between functional and effective connectivity (Friston, 2011). The interpretations of PPI are constrained by the choice of seeds and the hypothesized role(s) ascribed to the seed. Toward that end, our choice of the dACC was motivated by its role in cognitive control of brain networks (Carter et al., 1999; Paus, 2001; Bakshi et al., 2011).

The dACC plays an integral role in tasks of explicit cognitive control including response conflict and response monitoring (Braver et al., 2001; van Veen et al., 2001), and choice selection (Eshel et al., 2007). The dACC may serve to amplify task-relevant signals to heteromodal association regions of the cortex (Egner and Hirsch, 2005; Sohn et al., 2007). Thus, control processes from the dACC may influence the activity of core working memory systems, and the degree of this modulation may reflect the efficiency of interaction between control and working memory systems. Increased control-related modulation in part reflects decreased efficiency. This hyper-modulation by the dACC may strongly suggest inefficient control-related network profiles in OCD. These effects are again consistent with observed evidence in other disorders, for example, in the schizophrenia spectrum where increased dACC related modulation is strongly associated with the illness and risk for the illness (Bakshi et al., 2011). The absence of a parametric effect on dACC modulation appears related to highly increased aberrant dACC modulation at the lower level of demand in OCD: no intra-group increases in dACC modulation were observed in OCD as memory load increases. As such, the network effects constitute complementary signatures of FSTC dysfunction in OCD.

## Limitations and Conclusion

Brain network profiles will constitute an important frontier in the search for mechanisms and endophenotypes, and their evidence is an important expression of the goals advocated by Research Domain Criteria (RDoC: Insel et al., 2010). While our sample size (though small) is comparable to several other published studies, it nevertheless precludes us from assessing the role of co-morbid diagnoses and medication effects within OCD youth. These are important clinical questions, and an expansion of this sample is ongoing, and may permit more detailed assessment of our observed effects.

The specific neurochemical and molecular bases of these effects are obscured by the interpretational limits of both the fMRI signal (Logothetis, 2008) that cannot distinguish between a multiplicity of neuronal contributions to the hemodynamic response, and by the relatively limited class of inferences that can be drawn from the application of PPI analyses (Stephan, 2004). Moreover, these technical challenges are compounded by the fundamental limitation in understanding the correlates of brain structure and function: the fact that functional characteristics of brain networks exist in a regressive relationship with their structural substrates (Park and Friston, 2013). Thus, the same underlying structural networks can give rise to a multiplicity of functions and dysfunctions. Nevertheless, our results (and other studies we have cited) promise to reveal mechanisms of disease-related dysfunction as expressed in brain profiles. An understanding of putative mechanisms is a necessary precursor of treatment and cure. Therefore we propose that studies such as ours (and future extensions) will provide better elucidation of disease mechanisms than currently exist.

## Conflict of Interest Statement

The authors declare that the research was conducted in the absence of any commercial or financial relationships that could be construed as a potential conflict of interest.

## Abbreviations

dACC, dorsal anterior cingulate cortex; FSTC, frontal striatal thalamic circuits; OCD, obsessive-compulsive disorder; PPI, psychophysiological interaction.
